# Gesundheitsberichterstattung im Rahmen von Public Health Surveillance: Das Beispiel Diabetes

**DOI:** 10.1007/s00103-020-03201-z

**Published:** 2020-08-19

**Authors:** Lukas Reitzle, Rebecca Paprott, Francesca Färber, Christin Heidemann, Christian Schmidt, Roma Thamm, Christa Scheidt-Nave, Thomas Ziese

**Affiliations:** Abteilung für Epidemiologie und Gesundheitsmonitoring, Robert Koch-Institut (RKI), General-Pape-Str. 62–66, 12101 Berlin, Deutschland

**Keywords:** Surveillance, Diabetes mellitus, Gesundheitsberichterstattung, Dissemination, Umsetzung, Surveillance, Diabetes mellitus, Health reporting, Dissemination, Translation

## Abstract

Eine Kernaufgabe von Public Health ist die kontinuierliche Erfassung und Analyse von gesundheitsbezogenen Daten zu relevanten Krankheiten (Surveillance). Sie dient der zeitnahen Umsetzung von Maßnahmen zum Schutz der Gesundheit in der Bevölkerung. Dafür müssen relevante Informationen zur richtigen Zeit und in geeigneter Weise für die entscheidenden Zielgruppen bereitgestellt werden (Dissemination).

Eine Disseminationsstrategie unterstützt die effektive Ergebniskommunikation und berücksichtigt 3 Kernfragen: (1) „Was sind die relevanten Inhalte der Surveillance?“, (2) „Wer benötigt welche Informationen?“ und (3) „Wie werden die Ergebnisse den Zielgruppen bereitgestellt?“ Die Digitalisierung eröffnet hierbei neue Möglichkeiten für die Gestaltung der Formate.

Seit 2015 wird am Robert Koch-Institut die Diabetes-Surveillance aufgebaut. In einem strukturierten Konsensprozess wurden 4 gesundheitspolitisch relevante Handlungsfelder mit 40 Kennzahlen (Indikatoren) definiert. Anschließend wurden gemeinsam mit dem wissenschaftlichen Projektbeirat unter Berücksichtigung neuer Möglichkeiten durch die Digitalisierung erste Publikationsformate erarbeitet. Neben Artikeln in Fachzeitschriften stellen der Bericht „Diabetes in Deutschland“ und eine Webseite mit interaktiver Visualisierung der Ergebnisse die wichtigsten Formate der ersten Projektphase dar. Begleitend werden Twitter und Youtube für die Erhöhung der Reichweite genutzt.

In der nächsten Projektphase steht neben der Weiterentwicklung des Indikatorensets der Ausbau der Dissemination hin zu einer nutzer- und handlungsorientierten Berichterstattung im Mittelpunkt. In engem Austausch mit dem wissenschaftlichen Beirat sollen Anforderungen der Zielgruppen erfasst und in der Entwicklung weiterer Formate berücksichtigt werden.

## Abstract (en)

The continuous collection and analysis of health data on relevant diseases (surveillance) is at the core of public health. The surveillance enables the implementation of measures to protect the populations’ health. Therefore, relevant information needs to be provided in a timely and target-group-specific manner to the respective stakeholders.

A dissemination strategy supports the effective communication of results and considers three key questions: (1) “What content is relevant to the surveillance?”, (2) “Who requires which information?” and (3) “How are the results disseminated to the target audience?” In this context, digitalisation allows for novel possibilities in the design of publication formats.

Since 2015, diabetes surveillance has been established at the Robert Koch Institute. Within a structured process of consensus, we defined four fields of action relevant for health policy including 40 indicators. Thereafter, we developed the first publication formats in collaboration with the scientific advisory board of the project that reflected novel possibilities offered by digitalisation. In addition to articles in scientific journals, the essential formats of the first project phase comprise the report “Diabetes in Germany” and a website including interactive visualisations of results. Additional posts on Twitter and YouTube are used to increase coverage.

In addition to the further development of the indicator set, the focus of the next project phase is the advancement of the dissemination towards user- and action-oriented reporting. In close exchange with the scientific advisory board, we aim to explore the requirements of the target audience and reflect them in the design of further publication formats.

## Hintergrund – Public Health Surveillance

**Figure 1:**
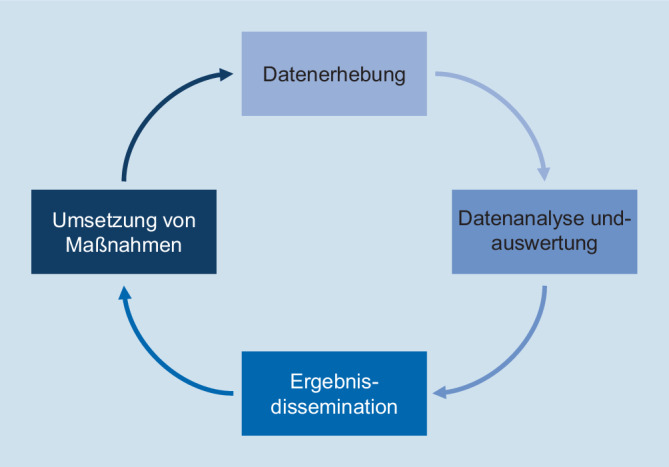

Public Health Surveillance wird von der Weltgesundheitsorganisation (WHO) definiert als kontinuierliche und systematische Erhebung, Zusammenführung und Analyse von gesundheitsbezogenen Daten und die zeitnahe Bereitstellung von Informationen an Entscheidungsträgerinnen und Entscheidungsträger als Grundlage für die Planung, Umsetzung und Evaluation von Public-Health-Maßnahmen ([1–3]; Abb. 1). Die Surveillance ist für den Bereich der akuten Gefahren durch Infektionskrankheiten bereits lange etabliert, hat jedoch in den letzten Jahren international auch große Bedeutung für den Bereich der nichtübertragbaren Krankheiten (Noncommunicable Diseases, NCD) erlangt [2, 4, 5].

Am Robert Koch-Institut (RKI) sind in den letzten Jahren Surveillance-Aktivitäten zum Schutz der öffentlichen Gesundheit daher auf einige nichtübertragbare Krankheiten und wichtige gemeinsame Risikofaktoren ausgedehnt worden. Im Fokus stehen dabei Krankheiten, die in Deutschland, aber auch weltweit zu den Haupttodesursachen zählen und erheblich zur Einschränkung der in Gesundheit verbrachten Lebenszeit beitragen. Nach Daten der Studie „Global Burden of Disease“ gilt dies in erster Linie für Herz-Kreislauf-Erkrankungen, Krebserkrankungen, chronische Atemwegserkrankungen, Diabetes mellitus und psychische Störungen [6, 7]. Die Prävention dieser Krankheiten durch die Reduktion wichtiger gemeinsamer Risikofaktoren unter Berücksichtigung zugrunde liegender sozialer Determinanten war bereits Gegenstand des 2013 verabschiedeten globalen WHO-Aktionsplans zu nichtübertragbaren Krankheiten [8]. Die Ziele des Aktionsplans wurden in insgesamt 3 Konferenzen der Vereinten Nationen (UN) weiterentwickelt und in die Agenda 2030 zu globalen Nachhaltigkeitszielen (Sustainable Development Goals, SDGs) integriert [8]. Das zentrale Nachhaltigkeitsziel „Gesundheit und Wohlbefinden für alle“ hat die Reduktion der vorzeitigen Sterblichkeit vor dem 70. Lebensjahr zum Ziel. Dies soll durch eine verbesserte Primärprävention wichtiger verhaltensbasierter Risikofaktoren, wie Alkohol- und Tabakkonsum, einer Sicherstellung des Zugangs zu medizinischer Versorgung und einer Förderung psychischer Gesundheit erreicht werden [9]. Dabei steht das Nachhaltigkeitsziel zur Gesundheit in Verbindung mit den anderen Nachhaltigkeitszielen, beispielsweise zur Bildung, zur Bekämpfung von Armut und zum Erhalt sicherer Lebenswelten, und verdeutlicht den Health-in-All-Policies(HiAP)-Ansatz zur Gesundheitsförderung und Prävention.

Seit Ende 2015 bis 2021 wird durch das Bundesministerium für Gesundheit (BMG) ein Forschungsprojekt zum Aufbau einer Diabetes-Surveillance als Beispiel einer umfassenden Public Health Surveillance für nichtübertragbare Krankheiten am RKI gefördert (www.diabsurv.rki.de; [9, 10]). Weiterhin fördert das BMG derzeit den Aufbau einer Mental Health Surveillance (2019–2021; www.rki.de/mhs) und ein Vorhaben zur Surveillance relevanter Einflussfaktoren auf die Entwicklung von Adipositas im Kleinkind- und Schulalter (2015–2020; www.rki.de/adimon). Zudem besteht bereits ein registerbasiertes System zur epidemiologischen Surveillance des Krebsgeschehens (www.krebsdaten.de). Für die jeweiligen Vorhaben stellt neben der Erhebung, Zusammenführung und Analyse von geeigneten Daten insbesondere die Dissemination der Ergebnisse eine Herausforderung dar. Dissemination bedeutet dabei, die gewonnenen Informationen zeitnah und adressatengerecht für relevante Akteure in Gesundheitspolitik, Forschung und Praxis als Entscheidungshilfe für eine verbesserte Planung, Umsetzung und Evaluation von Maßnahmen zum Schutz und zur Förderung der Gesundheit der Bevölkerung zur Verfügung zu stellen.

Allerdings wird bei der Dissemination wissenschaftlicher Forschungsergebnisse häufig das Phänomen des sogenannten „Translation Gap“ beobachtet, welcher den fehlenden oder deutlich verzögerten Eingang von Informationen aus der Wissenschaft in politische Entscheidungsprozesse sowie die Entwicklung von Maßnahmen beschreibt [11]. Studien aus den USA und UK haben gezeigt, dass wissenschaftliche Ergebnisse vorrangig in akademischen Fachzeitschriften in entsprechend akademischer Sprache veröffentlicht, jedoch nur selten in adressatengerechte Sprache und Formate übersetzt und der Politik aktiv in adäquater Form präsentiert werden [12, 13]. Daher sind die frühzeitige Planung und Entwicklung einer Strategie zur Dissemination der Ergebnisse der Surveillance von großer Bedeutung. Im Folgenden werden wissenschaftliche Grundlagen einer Disseminationsstrategie, neue Möglichkeiten für die Dissemination durch die Digitalisierung und abschließend die Umsetzung im Rahmen der Diabetes-Surveillance dargestellt.

## Disseminationsstrategie zur adressatengerechten Kommunikation

**Figure 2:**
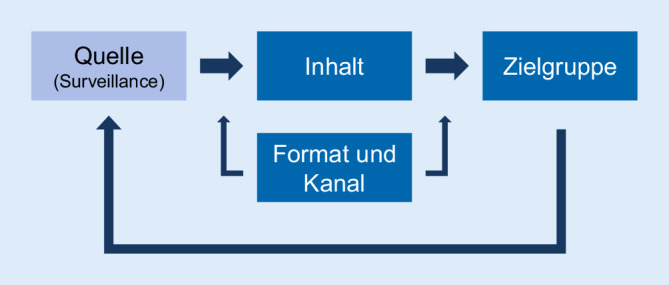

Für eine Disseminationsstrategie sind verschiedene Rahmenkonzepte aus den Kommunikationswissenschaften von Bedeutung [14]. Die häufigsten Konzepte im Bereich Public Health stellen „persuasive Kommunikation“, „Social Marketing“ und die „Diffusionstheorie“ dar. Bestandteil aller Konzepte sind 3 Kernfragen, welche in der Entwicklung einer Disseminationsstrategie einer Surveillance berücksichtigt werden sollen: (1) „Was sind die relevanten Inhalte der Surveillance?“, (2) „Wer benötigt welche Informationen?“ und (3) „Wie werden die Ergebnisse den Zielgruppen bereitgestellt?“ ([15, 16]; Abb. 2).

Angewandt auf die Surveillance nichtübertragbarer Erkrankungen wie Diabetes mellitus sollen zunächst die relevanten Themen zur Abbildung der Krankheitsdynamik identifiziert werden, welche für die Gesundheitspolitik und die Entwicklung von Maßnahmen notwendig sind. Die Themen sollen dabei nicht allein auf die Prävalenz, Inzidenz, Mortalität und Versorgung der Erkrankung fokussieren, sondern auch ihre relevanten Determinanten im Blick behalten [17]. Hierbei sind sowohl verhaltensbasierte Risiko- und Schutzfaktoren als auch soziale und verhältnisbasierte Determinanten zu berücksichtigen.

Daran anknüpfend sollen die relevanten Zielgruppen aus den Bereichen Gesundheitspolitik, Versorgung und Forschung und deren spezifischer Informationsbedarf („need for information“) analysiert werden [16]. Die Zielgruppen sollen mittels spezifischer Formate angesprochen werden, in denen Sprache, visuelle Ausgestaltung sowie die ausgewählten Inhalte den jeweiligen Anforderungen entsprechen. Hierbei ist es besonders wichtig, den aktiven Austausch mit den Zielgruppen zu suchen und nachhaltige Netzwerke zu schaffen. Denn die partizipative Entwicklung der Disseminationsstrategie und der regelmäßige Austausch erhöhen die Akzeptanz und die Nutzung der Ergebnisse bei den Zielgruppen [15, 18].

Der Gesundheitspolitik kommt als Zielgruppe der Surveillance eine besondere Bedeutung zu, da diese maßgeblich zur Umsetzung von Maßnahmen zum Schutz und zur Förderung der Gesundheit beiträgt. Oliver et al. haben in ihrer Übersichtsarbeit verschiedene begünstigende und hemmende Faktoren für den Transfer von wissenschaftlichen Ergebnissen zu politischen Entscheidungsträgerinnen und -trägern berichtet [19]. Die wichtigsten begünstigenden Faktoren waren die Verfügbarkeit und der Zugang zu den Daten sowie deren Klarheit, Relevanz und Verlässlichkeit. Umgekehrt stellten die gleichen Faktoren auch die größten Hemmnisse dar, sofern sie nicht erfüllt waren. Im Besonderen zeigte sich auch hier, dass die enge Zusammenarbeit und die Beziehung zwischen Politik und Wissenschaft eine fördernde Wirkung auf die Verwendung von wissenschaftlichen Erkenntnissen als Grundlage für gesundheitspolitische Entscheidungen hat [19].

Um die Disseminationsstrategie kontinuierlich weiterzuentwickeln, ist eine regelmäßige Evaluation der Ergebnisnutzung unerlässlich. Hierbei können kurzfristige Maßzahlen wie die Anzahl von Downloads und Zitationen sowie eine zusätzliche Erfassung der Social-Media-Aktivitäten mittels Altmetriken zum Einsatz kommen [15]. Weiterhin kann die Nutzung der Ergebnisse in der Erstellung von Leitlinien und gesundheitspolitischen Entscheidungen analysiert werden. Auch hierbei kann der direkte Dialog mit den Zielgruppen entscheidend dazu beitragen, die Nutzung der Surveillance-Informationen zu fördern [15, 17].

## Bedeutung der Digitalisierung für die Dissemination

### Neue Möglichkeiten der Datengewinnung

Wesentliche Datengrundlagen der Gesundheitsberichterstattung werden in der Onlinedatenbank „Informationssystem der Gesundheitsberichterstattung“ (IS-GBE) bereitgestellt, welche seit 1999 gemeinsam vom Statistischen Bundesamt und RKI getragen wird (www.gbe-bund.de). Seit seiner Einführung hat sich der Datenpool der IS-GBE sukzessive um zahlreiche nationale und internationale Indikatoren erweitert und ist fester Bestandteil in den Formaten der Berichterstattung des Bundes und der Länder [20]. Dabei werden sowohl Informationen aus Primärdaten wie den Befragungs- und Untersuchungssurveys des RKI als auch Informationen aus amtlichen Statistiken und weiteren nationalen wie internationalen Daten (z. B. WHO, OECD) vorgehalten. Die Datenbank stellt aggregierte Ergebnismengen zur Verfügung, die nach vorgegebenen Merkmalen stratifizierbar sind, allerdings keinen Rückgriff auf die zugrunde liegenden Individualdaten erlauben.

In den letzten Jahren wurden neue technische und rechtliche Möglichkeiten geschaffen, die insbesondere die personenbezogenen Routinedaten der Sozialversicherung für die Gesundheitsberichterstattung nutzbar machen [21]. Folgerichtig wird im Datenmodell der Diabetes-Surveillance neben den etablierten Surveys des RKI und den seit 2017 neu am RKI eingeführten Ad-hoc-Studien [22] ebenfalls auf Routinedaten der Sozialversicherung zurückgegriffen [10, 23]. Eine besondere Rolle nehmen hierbei die Daten der Gesetzlichen Krankenversicherung (GKV) nach der Datentransparenzverordnung (DaTraV) ein, die seit dem Jahr 2014 auf Antrag beim Deutschen Institut für Medizinische Dokumentation und Information (DIMDI) verfügbar sind [24]. Seit 2020 ist die Datenaufbereitungsstelle des DIMDI in das Bundesinstitut für Arzneimittel und Medizinprodukte (BfArM) eingegliedert und wird auf Grundlage einer novellierten DaTraV zu einem Forschungsdatenzentrum (FDZ) weiterentwickelt. Im Unterschied zu Daten einzelner Krankenkassen sind in den DaTraV-Daten keine historisch gewachsenen Populationen mit zwischen den Kassen abweichender Alters- und Sozialstruktur vorhanden und langjährige Verlaufsbetrachtungen unter Erhalt des Personenbezugs sind möglich. Hierdurch werden die typischen Selektionseffekte (Kassenbias) vermieden und die Ergebnisse besitzen Gültigkeit für die gesamte GKV-Population, welche circa 90 % der Bevölkerung Deutschlands einschließt. Außerdem ist eine detaillierte regionale Darstellung möglich, welche besonders für die kommunale und regionale Gesundheitspolitik relevant ist. Perspektivisch sollen die derzeit bestehenden Nachteile, die insbesondere die Aktualität und den Zugang zu den Daten betreffen, auf Grundlage des im Jahr 2019 verabschiedeten Gesetzes für eine bessere Versorgung durch Digitalisierung und Innovation (Digitale-Versorgung-Gesetz – DVG) abgebaut werden [25].

### Neue Möglichkeiten der Visualisierung

**Figure 3:**
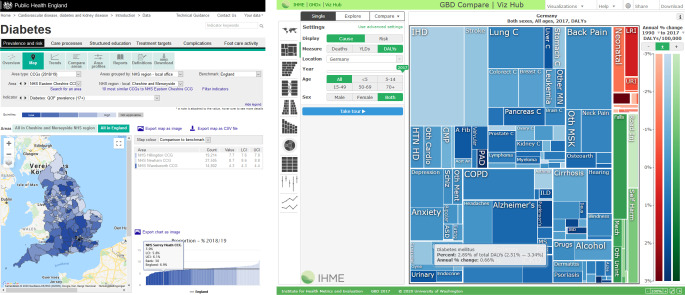

Die grafische Aufbereitung kann den Zugang und die Nutzung von Ergebnissen der Surveillance maßgeblich fördern [17, 26]. Wie in anderen Fachbereichen eröffnet die Digitalisierung auch im Bereich der Epidemiologie und Public Health neue Möglichkeiten zur Analyse und Visualisierung von komplexen Datensätzen [27]. Insbesondere können Visualisierungen dabei unterstützen, große Ergebnismengen zugänglich zu machen, und es den Nutzerinnen und Nutzern erlauben, automatisiert Teilmengen für relevante Fragestellungen abzufragen und zu analysieren [28]. In der Epidemiologie von infektiösen Erkrankungen werden Visualisierungs- und Analysetools schon für vielfältige Fragestellungen genutzt [29]. Beispielsweise konnte im Projekt „AIDSVu“ die Darstellung regionalisierter Daten zu HIV-Infektionen in den USA in interaktiven Karten die Entwicklung von spezifischen Maßnahmen in betroffenen Regionen fördern [30]. Aber auch im Bereich der nichtübertragbaren Erkrankungen ermöglichen Visualisierungstools wie „GBD Compare“ des Institute for Health Metrics and Evaluation (USA; [6]) oder das „Fingertips Tool“ von Public Health England [31] einen interaktiven Datenzugang (Abb. 3). Beispielsweise ermöglicht das „Fingertips Tool“ die Abfrage von verschiedenen Indikatoren zu Diabetes wie der Prävalenz auf regionaler Ebene, welche von der kommunalen Politik zur Versorgungsplanung genutzt werden können. Essenziell dabei ist die Beschreibung der Qualität und der Limitationen der Daten, sogenannter Metadaten, damit die Nutzerinnen und Nutzer in die Lage versetzt werden, die Ergebnisse richtig einordnen und interpretieren zu können [28].

### Neue Möglichkeiten der Kommunikation

Die Verbreitung von Ergebnissen der Public-Health-Forschung über verschiedene Presse- und Medienkanäle kann dazu beitragen, eine erhöhte Aufmerksamkeit für das Thema zu erzeugen und das Thema auf die gesundheitspolitische Tagesordnung zu setzen [32]. Im Zuge der Digitalisierung ergeben sich mit der Etablierung sozialer Medien zusätzlich neue Wege zur Verbreitung [33]. Soziale Medien schaffen Kanäle zur Kommunikation und Vernetzung über das Internet und ermöglichen einer Vielzahl von Nutzenden das Generieren, Teilen, Empfangen und Kommentieren von Inhalten [34].

Für die Gesundheitskommunikation können Plattformen wie Facebook, Twitter oder Youtube gemäß einer systematischen Übersichtsarbeit [34] auch im Bereich von Public Health Surveillance bestimmte Vorteile bieten: Sie erlauben es, zeitnah, kostengünstig und leicht zugänglich Informationen zur Verfügung zu stellen und das Bewusstsein für public-health-relevante Themen zu steigern. Soziale Medien erhöhen die Verfügbarkeit gesundheitsbezogener Informationen und durch den interaktiven Austausch von Nutzenden in bestehenden Netzwerken kann ein breites Publikum erreicht werden. Inhalte können auf verschiedene Zielgruppen zugeschnitten werden und auch Zielgruppen erreichen, die durch andere Formate nicht in gleichem Maße angesprochen werden.

In der Public-Health-Praxis werden soziale Medien bereits häufig dazu genutzt, um über relevante Gesundheitsthemen aufzuklären (z. B. Centers for Disease Control and Prevention [CDC] zu Diabetes auf Twitter und Facebook [35]) oder die Kommunikation in Krisenlagen zu unterstützen (z. B. Bundeszentrale für gesundheitliche Aufklärung (BZgA) zum neuartigen Coronavirus (SARS-CoV-2) auf Youtube [36]). Public-Health-Organisationen verwenden soziale Medien zunehmend auch ergänzend zu bestehenden Strategien zur Dissemination ihrer Forschungsergebnisse. Jedoch besteht in vielen Fällen noch nennenswerte Unsicherheit darüber, wie ihr Einsatz am effektivsten zur besseren Umsetzung evidenzbasierter Forschung beitragen kann [33]. Zur Klärung der offenen Fragen ist eine begleitende Evaluation von zentraler Bedeutung [36]. Dabei sollten die Möglichkeiten sozialer Medien zur aktiven Ansprache und Partizipation der Zielgruppen bei der Ergebnisdissemination stärkere Berücksichtigung finden [37].

## Das Beispiel Diabetes-Surveillance

Wie eingangs erwähnt, wird im Hinblick auf die Entwicklung einer Surveillance von nichtübertragbaren Erkrankungen in Deutschland seit 2015 das Forschungsprojekt „Aufbau einer Nationalen Diabetes-Surveillance“ am RKI gefördert. Das Projekt wird von einem wissenschaftlichen Beirat mit Vertreterinnen und Vertretern aus den Bereichen Politik, Forschung, Versorgung sowie Patientinnen- und Patientenvertretung begleitet. Primäres Ziel der Diabetes-Surveillance ist es, ein indikatorenbasiertes Surveillance-System für Diabetes zu implementieren, welches kontinuierlich verlässliche Daten zur Unterstützung der Entwicklung von Maßnahmen zum Schutz vor Diabetes und zur Verbesserung der Versorgung von Menschen mit Diabetes liefert. In der ersten Projektphase von 2015 bis 2019 wurde das Rahmenkonzept erarbeitet, wurden Indikatoren definiert, Datenquellen erschlossen und erste Formate zur Dissemination entwickelt. Neben der Aktualisierung des Indikatorensets und dem Ausbau der Datengrundlage der Diabetes-Surveillance steht in der zweiten Projektphase (2020 bis 2021) die Weiterentwicklung der Dissemination zu einer nutzer- und handlungsorientierten Berichterstattung im Fokus. Im Folgenden werden das erarbeitete Konzept zur Dissemination und die in der ersten Projektphase erstellten Formate vorgestellt.

### Was sind die relevanten Inhalte?

Im ersten Schritt wurde in enger Abstimmung mit dem wissenschaftlichen Beirat ein Rahmenkonzept erarbeitet, welches in Anlehnung an das Nationale Gesundheitsziel „Diabetes mellitus Typ 2“ [37] relevante Handlungsfelder enthält: (1) Diabetesrisiko reduzieren, (2) Diabetesfrüherkennung und -behandlung verbessern, (3) Diabeteskomplikationen reduzieren und (4) Krankheitslast und Krankheitskosten senken [10]. Kernbestandteil des Konzepts sind gesundheitspolitisch relevante, messbare Indikatoren, welche das Krankheitsgeschehen von Diabetes abbilden. Nach Literaturrecherche zu bestehenden internationalen und nationalen Indikatorensystemen zu Diabetes und nichtübertragbaren Erkrankungen wurden in einem strukturierten Konsensprozess 40 Indikatoren beziehungsweise Indikatorengruppen für die Abbildung der Krankheitsdynamik des Diabetes ausgewählt und den 4 Handlungsfeldern zugeordnet [10].

Zur Implementierung der ausgewählten Indikatoren wurden mögliche Datenquellen gesichtet. Neben den Primärdaten der bundesweiten Gesundheitssurveys des RKI als wichtige Datenquellen wurde die Bedeutung von Sekundärdaten für die Diabetes-Surveillance diskutiert und die Umsetzbarkeit ihrer Nutzung in verschiedenen Kooperationsprojekten geprüft [23, 24]. Bei der Auswahl der Datenquellen ist neben der Datenqualität auch relevant, ob diese regelmäßig zur Verfügung stehen, ob sich Zeitreihen abbilden lassen und ob die Daten eine Stratifizierung nach Region und sozialen Determinanten wie Bildung zulassen. Im Ergebnis werden derzeit 14 Indikatoren aus Primärdaten und 14 Indikatoren aus Sekundärdaten (davon 7 Indikatoren aus DaTraV-Daten [38]) abgebildet; 12 Indikatoren benötigen Informationen aus beiden Datenquellen (davon 7 Indikatoren mit Einbezug von DaTraV-Daten).

### Wer benötigt welche Informationen?

Bei der Frage nach den Zielgruppen orientiert sich die Surveillance am Konzept der Gesundheitsberichterstattung des Bundes am RKI, worin verschiedene Zielgruppen benannt sind, darunter Politik, Akteure im Gesundheitswesen, Forschung und Lehre, Studierende, Fachöffentlichkeit sowie Bürgerinnen und Bürger [20]. Da die primären Ziele der Diabetes-Surveillance die Unterstützung der Entwicklung von Maßnahmen zum Schutz vor Diabetes und die Verbesserung der Versorgung von Menschen mit Diabetes sind, stehen die Politik und die verschiedenen Akteure im Gesundheitswesen als Zielgruppen im Mittelpunkt und wurden in der ersten Projektphase priorisiert.

Hierzu wurden der wissenschaftliche Beirat sowie Vertreterinnen und Vertreter aus dem BMG eng in die Entwicklung des ersten Konzepts einer Disseminationsstrategie einbezogen. Gemeinsam wurden erste Formate zur deskriptiven Ergebnisdarstellung entwickelt und die Inhalte in Diskussion mit dem Beirat priorisiert. Hierbei wurden bekannte Faktoren, die einen Wissenstransfer fördern, wie Klarheit, Relevanz und Verlässlichkeit der Ergebnisse sowie deren Verfügbarkeit in der Erstellung berücksichtigt. Die Verlässlichkeit der Ergebnisse aus den Datenquellen wurde auf Anregung des Beirats in einem Absatz zur Datenqualität zu jedem Indikator im Bericht und auf der Webseite klar benannt und die Methodik ausführlich beschrieben. Im Konsens wurden die Ergebnisse eingeordnet, um die Relevanz unter Berücksichtigung von bestehenden Limitationen detailliert zu erläutern. Um der Verfügbarkeit gerecht zu werden, sind alle entwickelten Formate wie der Diabetesbericht und die Webseite ohne Barrieren über das Internet zugänglich (www.diabsurv.rki.de).

Da in Deutschland neben der Bundesebene auch weitreichende gesundheitspolitische Zuständigkeiten auf der Länderebene verortet sind, findet sich hier ebenfalls eine wichtige Zielgruppe der Diabetes-Surveillance. Um diese Zielgruppe bei der Aufbereitung der Informationen zu berücksichtigen, ist zum einen die Gesundheitsberichterstattung der Länder im wissenschaftlichen Beirat der Diabetes-Surveillance vertreten. Zum anderen wurden die Anforderungen der Länder an die Diabetes-Surveillance und deren Disseminationsstrategie in einem gemeinsamen Workshop erörtert [39]. Als Ziel wurde vereinbart, die Indikatoren auch regionalisiert nach Bundesland darzustellen, soweit ausreichende Fallzahlen vorhanden sind.

Weiterhin befindet sich die Diabetes-Surveillance in engem Austausch mit der BZgA, welche mit dem Projekt „Nationale Aufklärungs- und Kommunikationsstrategie zu Diabetes mellitus“ das Ziel hat, Aufklärungs- und Informationsangebote für die Allgemeinbevölkerung und für Menschen mit Diabetes zielgruppengerecht bereitzustellen [40]. Gemeinsam wurde von RKI und BZgA eine Ad-hoc-Studie bei Erwachsenen mit und ohne Diabetes durchgeführt, in der beispielsweise die Informationsbedarfe bezüglich Diabetes repräsentativ für die Bevölkerung erhoben wurden [41]. Initiiert von der BZgA wurde das Diabetesinformationsportal www.diabinfo.de erstellt, welches gemeinsam vom Deutschen Diabetes-Zentrum (DDZ), dem Deutschen Zentrum für Diabetesforschung (DZD) und dem Helmholtz Zentrum München getragen wird. Die epidemiologischen Ergebnisse der Diabetes-Surveillance fließen in diese Plattform ein.

Weitere Zielgruppen werden derzeit nicht in gleichem Maße aktiv angesprochen. Die Webseite ermöglicht einfachen und transparenten Zugang zu allen Ergebnissen und diese können beispielsweise von Studierenden und Lehrenden im Bereich Public Health verwendet werden. Darüber hinaus werden methodische und konzeptionelle Erkenntnisse in der Etablierung der Surveillance und der Erschließung neuer Datenquellen veröffentlicht, die für Forschende im Bereich Public Health relevant sind.

### Wie werden die Ergebnisse den Zielgruppen bereitgestellt?

**Figure 4:**
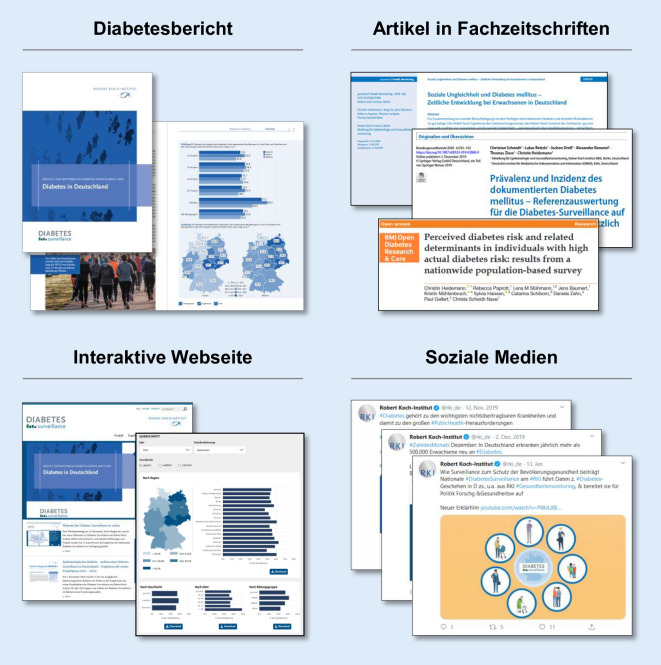

Um die Formate für die Dissemination der Diabetes-Surveillance zu entwickeln, wurden in einem internationalen Workshop Best-Practice-Beispiele aus der Schweiz und England vorgestellt [42]. Darüber hinaus erfolgten eine Onlinebefragung und eine Internetrecherche zur Erfassung der verwendeten Formate für die Gesundheitsberichterstattung zu Diabetes und anderen nichtübertragbaren Erkrankungen innerhalb der Mitgliedsstaaten der Europäischen Union und der Organisation für wirtschaftliche Zusammenarbeit und Entwicklung (OECD; [43]). Basierend auf dieser Grundlage wurden gemeinsam mit dem wissenschaftlichen Beirat die ersten 4 Formate entwickelt (Abb. 4), die den Startpunkt für die Disseminationsstrategie bilden.

Erstens wurde in Abstimmung mit dem Wissenschaftlichen Beirat ein Diabetesbericht erarbeitet, der sich primär an die Gesundheitspolitik und Akteure im Gesundheitswesen richtet [44]. Hierin sind die Kernergebnisse der Diabetes-Surveillance dargestellt. Bei der Erstellung des Berichts wurde auf eine einheitliche und klare Struktur geachtet. Die Kapitel orientieren sich an den 4 Handlungsfeldern und bestehen im Kern aus zweiseitigen Faktenblättern zu ausgewählten Indikatoren. Einleitend findet sich jeweils eine Zusammenfassung, welche einen prägnanten Überblick über die Entwicklungen im jeweiligen Handlungsfeld liefert. Ein Ausblick rundet den Bericht ab. Neben klar und verständlich dargestellten Ergebnissen benennt der Bericht auch Datenlücken und Forschungsbedarfe, wenn bisher keine ausreichende Datengrundlage zur Verfügung steht.

Zweitens wurde eine Webseite für die Diabetes-Surveillance entwickelt (www.diabsurv.rki.de). Diese bietet einen Zugang zu allen Ergebnissen der Surveillance mit zusätzlichen Stratifizierungsmöglichkeiten über den Bericht hinaus sowie der Darstellung von altersstandardisierten Werten im zeitlichen Verlauf. Interaktive Visualisierungen fördern das Verständnis der Daten. Ausführliche Beschreibungen der Datenquellen inklusive deren Qualität und Limitationen helfen bei der Einordnung. Ergänzende Links geben schnellen Zugriff auf weiterführende Informationen zu Datenquellen und Publikationen in wissenschaftlichen Fachzeitschriften. Darüber hinaus enthält die Webseite aktuelle Informationen zum Projekt. Die Webseite stellt eine Anlaufstelle für alle Zielgruppen dar, richtet sich aber vor allem an Gruppen mit Vorwissen in den Bereichen Epidemiologie und Public Health.

Drittens dienen neben der Webseite auch Veröffentlichungen in nationalen und internationalen Fachzeitschriften und Präsentationen auf Fachtagungen dazu, die Ergebnisse der Wissenschaft zur Verfügung zu stellen und kritisch zu diskutieren. Zum einen werden neue Methoden, Datenquellen und Ergebnisse, wie beispielsweise die Schätzung der Inzidenz und Prävalenz des Diabetes auf Basis von Daten gesetzlich Krankenversicherter [38] oder die soziale Ungleichheit in Zusammenhang mit Diabetes, im Detail beleuchtet [45]. Zum anderen werden Konzepte zur Indikatorenauswahl und zum Aufbau der Diabetes-Surveillance publiziert [9, 46].

Schließlich sind zur Verbreitung der Surveillance-Ergebnisse und zur Steigerung der Aufmerksamkeit auch Presse und Medien wichtige Zielgruppen. Klassische Pressemitteilungen, die gleichzeitig mit Artikeln oder dem Diabetesbericht erscheinen, erhöhen deren Reichweite [47]. Zusätzlich werden im Rahmen der Diabetes-Surveillance auch die Potenziale der sozialen Medien genutzt und regelmäßige Twitter-Mitteilungen sollen die Bekanntheit der Diabetes-Surveillance fördern. Weiterhin wurde ein Erklärvideo zur Diabetes-Surveillance auf Youtube veröffentlicht [48], welches Zielgruppen über die Fachöffentlichkeit hinaus zu den Projektinhalten informiert.

## Fazit und Ausblick

Die Diabetes-Surveillance hat zum Ziel, eine valide Datengrundlage der Krankheitsdynamik für die Entwicklung von spezifischen Maßnahmen zum Schutz vor Diabetes und zur Förderung der Gesundheit von Menschen mit Diabetes zu schaffen. Ein wichtiger Faktor zur Förderung des Wissenstransfers von der Surveillance zur Politik und anderen Nutzenden ist der regelmäßige gemeinsame Austausch. Zusätzlich erleichtern eine zielgruppenspezifische Ausgestaltung der Formate ohne Zugangsbarrieren und eine klare Darstellung der Ergebnisse deren Nutzung.

Im Rahmen der ersten Projektphase wurden in Zusammenarbeit mit dem wissenschaftlichen Beirat die relevanten Themenfelder zu Diabetes identifiziert und erste Formate hin zu einer Disseminationsstrategie entwickelt. Dabei erfolgten auch die Bewertung und Einordnung der Ergebnisse unter Einbezug der Expertise des Beirats, um den Konsens hinsichtlich der Verlässlichkeit der Aussagen zu stützen. Die Dissemination der Ergebnisse umfasst klassische Formate wie einen Diabetesbericht, wissenschaftliche Artikel und Pressemitteilungen, nutzt aber auch neue technische Möglichkeiten zur interaktiven Visualisierung auf der Webseite und bindet soziale Medien zur Erhöhung der Reichweite in die Dissemination ein.

In der folgenden Projektphase der Diabetes-Surveillance soll zum einen das Indikatorsystem aktualisiert und dessen Datengrundlage ausgebaut werden. Zum anderen steht die Weiterentwicklung der Disseminationsstrategie in Zusammenarbeit mit dem wissenschaftlichen Beirat hin zu einer nutzer- und handlungsorientierten Berichterstattung im Fokus. Thematisch liegt dabei ein besonderes Augenmerk auf den verhältnisbasierten Faktoren sowie dem lebensphasenspezifischen Zugang. Die Nutzung bisheriger Formate soll evaluiert und die Anforderungen insbesondere der Politik und weiterer wichtiger Akteure im Gesundheitswesen erfasst werden. Es ist vorgesehen, dies im kontinuierlichen Dialog, beispielsweise in Workshops und Sitzungen des Wissenschaftlichen Beirats, fortzuführen.
